# Simple Lymphangioma to Generalized Lymphatic Anomaly: Role of Imaging in Disclosure of a Rare and Morbid Disease

**DOI:** 10.1155/2015/603859

**Published:** 2015-04-12

**Authors:** Manoj Joshi, Dilip S. Phansalkar

**Affiliations:** ^1^Department of Pediatric Surgery, Pondicherry Institute of Medical Sciences, Kalapet, Pondicherry 605014, India; ^2^Department of Radiology, Pondicherry Institute of Medical Sciences, Kalapet, Pondicherry 605014, India

## Abstract

Generalized lymphatic anomaly is a rare multisystem congenital disorder in which multiple organs are involved. Imaging features often overlap with other complex lymphatic anomalies and diagnosis is difficult. Treatment options are limited, not remedial and prognosis is poor. We report a 12-year-old male who presented with axillary and chest wall lymphangioma but was subsequently diagnosed as having diffuse lymphangiomatosis affecting lungs, liver, spleen, and bones on computerized tomography scan. We suggest complete radiological evaluation of susceptible adolescent children with lymphangioma to avoid diagnostic delay in this morbid condition. We also discuss radiological features of other similar complex lymphatic anomalies and crucial role of imaging in diagnosis.

## 1. Introduction

Generalized lymphatic anomaly (GLA), also earlier referred to as diffuse lymphangiomatosis, is a rare congenital lymphatic malformation originating from persistence of dilated lymphatics at 14–20th week of life. Approximately 65% of affected patients are infants and young children [1]. Prognosis is poor and major cause of morbidity and mortality is deterioration of lung function and infection [2]. Exact incidence of this condition is not known due to its rarity.

## 2. Case Presentation

**Figure 1:**
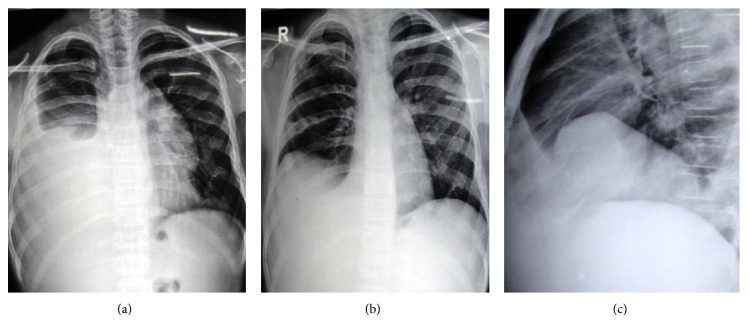
(a) Chest X-ray posteroanterior view showing right moderate pleural effusion with left mediastinal effusion (arrow). (b) Chest X-ray PA view postaspiration film showing residual right effusion and multiple osteolytic lesions in ribs bilaterally. (c) Chest X-ray right lateral view showing well defined opacity in lower part of chest.

**Figure 2:**
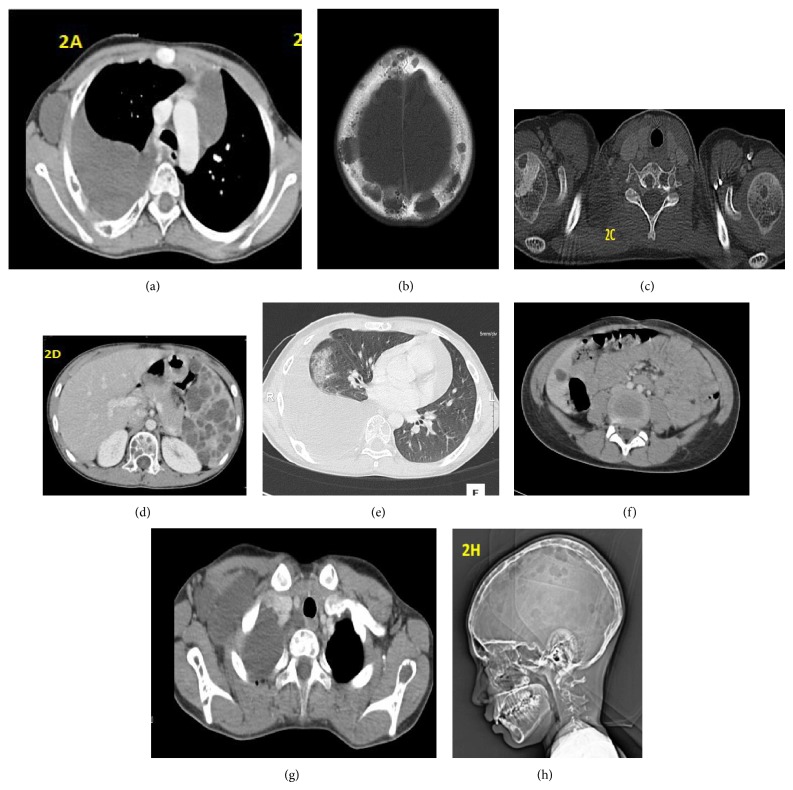
(a) CT scan chest (axial section) showing right pleural effusion, left mediastinal effusion, right chest wall hypodense lesion, and osteolytic lesions in ribs on right side. (b) CT scan skull (bony window) showing multiple osteolytic lesions without marginal sclerosis. (c) CT scan through shoulders (bony window) showing osteolytic lesions in head of each humerus without marginal sclerosis and vertebral body lesion with marginal sclerosis. (d) CT scan abdomen showing multiple cystic lesions in spleen with splenomegaly and osteolytic lesions in vertebra. (e) CT scan thorax (pulmonary window) showing pulmonary lymphangioma appearing as nonhomogenous consolidation with few small cystic hypodense shadows laterally with right pleural effusion. (f) CT scan abdomen showing hypodense lesion in liver. (g) CT scan of upper chest showing hypodense lesion in right axilla and pleural effusion with hypodense lesion in left scapula. (h) Skull topography showing multiple osteolytic “punched-out” lesions.

**Figure 3:**
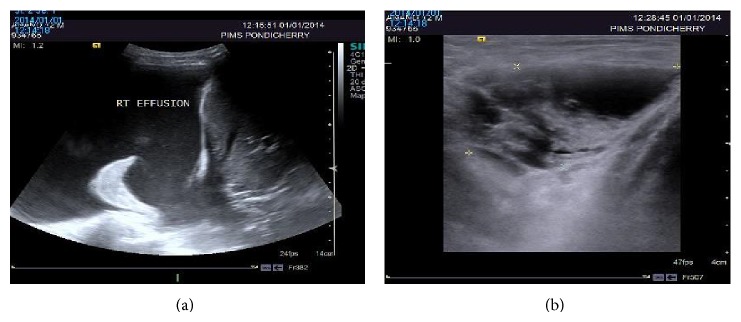
(a) Ultrasound examination of right lower chest showing right pleural effusion. (b) Ultrasound examination of soft tissue swelling in right axilla showing cystic mass with thick and thin septae due to lymphangioma.

A 12-year-old male presented with swellings over right axilla and supraclavicular region and difficulty in breathing on exertion since one month. He underwent excision of a supraclavicular swelling one year back at some other hospital. Biopsy report was suggestive of lymphatic cyst. He subsequently developed recurrence of swelling over operated site and right axilla. On examination, his vital signs were stable. A well circumscribed soft, nontender, and subcutaneous swelling was seen over right axilla and a diffuse swelling was seen over right side of the neck and anterior aspect of the lower chest. On auscultation air entry was grossly diminished on right side of chest. There were no added sounds. On abdominal examination, spleen was massively enlarged and firm. X-ray chest anteroposterior and lateral view and sonography were done. It was suggestive of gross right pleural effusion and left mediastinal opacity (Figures 3(a) and 1(a)). Sonography of right supraclavicular and axillary regions showed multiseptate cystic lesions, largest measuring being 3.6 × 2 centimeter (Figure 3(b)). Multiple therapeutic pleural fluid aspirations were done and sent for fluid analysis. Approximately 2.5−3 liters of fluid was drained in 4-5 days. It was nonchylous lymphatic fluid with high lymphocytic count. Culture was sterile. He symptomatically improved and there was no recollection of pleural fluid. Postaspiration chest X-ray however showed multiple osteolytic lesions over ribs and a well defined opacity on right side that is more prominent in lateral view (Figures 1(b) and 1(c)). Child was further evaluated with CT scan thorax. It revealed pleural effusion along with consolidation of lung on right side (Figure 2(e)). Hypodense lesions were noted along the left mediastinal margin and on right chest wall with multiple osteolytic lesions over ribs and vertebra (Figure 2(a)). Subsequently, sonography and contrast CT scan of abdomen, pelvis, and plain CT skull were performed. Reports revealed multiple splenic cysts, cystic lesion in liver, and multiple osteolytic lesions in skull vault bones, vertebral bodies, and upper end of each humerus (Figures 2(b), 2(c), 2(d), 2(f), and 2(h)). On the basis of these radiological findings he was diagnosed as having generalized lymphatic anomaly. Parents were counseled about the nature of condition and its prognosis. He was advised splenectomy because of risk of infections, spontaneous rupture, or trauma leading to hemoperitoneum. Patient was asymptomatic after four months of follow-up. But, later, parents decided against further treatment.

## 3. Discussion

Rodenberger first described this condition as generalized cystic lymphangiomatosis in 1828 [3]. Recently, it has been mentioned as generalized lymphatic anomaly in literature and its imaging features have been differentiated with other complex lymphatic anomalies, like Gorham-stout disease (GSD) and Kaposiform lymphangiomatosis (KLA) [4]. It is characterized by diffuse proliferation of lymphatic tissue in almost any organ of the body with exception of central nervous system [5]. Organs like lungs, liver, spleen, skull bone, long bones, and mandible may be involved [6]. Because of rare incidence in adolescent children, it is described as isolated case reports and short case series [3, 6–8].

Imaging features of GLA often overlap with KLA and GSD. It is reported that loss of cortical bone, contiguous involvement, and progressive osteolysis are key differentiating features of GSD from GLA [2]. In GLA, numbers of bones affected are more, with noncontiguous involvement, and round, osteolytic lesions often referred to as “punched-out.” In KLA there are multiple noncontiguous, lytic, and cortex sparing lesions. Thoracic involvement is much more in KLA as compared to GLA. Thickening along bronchovascular bundle and interlobular septal thickening is uncommonly seen in GLA [2]. KLA therefore is more aggressive disease with episodes of infection and hemorrhage in pleura and pericardium with incomplete recovery [2]. This overlapping imaging has led to hypothesis that KLA arises from GLA. Multiorgan lymphangiomatosis in CT scan and MRI is suggestive of the diagnosis of GLA [9]. Soft tissue involvement and bone marrow changes are better assessed by MRI.

Our case presented with simple subcutaneous lymphangioma and clinical evidence of effusion on right side. X-ray chest and spirometry confirmed massive right pleural effusion and reduced vital capacity due to restricted lung disease, respectively. It was initially suspected that this effusion was a complication of previous surgical procedure due to incomplete removal of lymphatic cyst in supraclavicular region. But after drainage of approximately 2.5 to 3 liters of nonchylous exudative pleural fluid, a well defined opacity was seen in posteroanterior and lateral view of chest X-ray. This was persisting even after repeated aspiration. This finding prompted us to perform CT scan thorax. It showed this hypodense area as mediastinal pleural effusion associated with involvement of lung parenchyma. There was no interlobular septal thickening or thickening along bronchovascular bundle seen on CT scan.

Bony lesions were noted on chest X-ray and CT scan involving ribs, skull, humerus, scapula, and vertebrae. They were osteolytic in the ribs with sharply defined margins. One differentiating feature noted in our case was mixed areas of lesions with and without marginal sclerosis. Usually lesions with marginal sclerosis are commonly reported. Lesions in GLA commonly involve ribs, skull vault, and extremity long bones. These lesions rarely resolve spontaneously. However, usually they lead to severe bone pain, pathological fractures, and joint deformity due to extreme osteolysis [2]. Bony lesions with mediastinal and pleural fluid collections usually carry poor prognosis. In our case, child had no symptoms related to bones.

It was interesting to note in our case that massive splenomegaly was detected during clinical examination in our patient and it was not a presenting complaint. Sonography and CT scan of the abdomen revealed multiple hypoechoic and hypodense lesions in the spleen, respectively. Contrast CT scan showed diffuse subcapsular nonenhancing cysts in spleen confirming splenic lymphangiomatosis. Radiological confirmation is therefore prudent for diagnosis of cystic involvement of nonpalpable spleen. Massive splenomegaly is at risk of trauma, infections, and spontaneous hemorrhage causing hemoperitoneum. Splenectomy is therefore advisable in such cases [3].

Role of surgery in GLA is limited. Options available for treatment are not curative and include repeated thoracocentesis, pericardiocentesis, or pleurodesis for recurrent pleural effusions. Recurrence rate is higher with these modalities. Though our follow-up was short, our case had no recurrence after repeated thoracocentesis. Thoracoscopic ligation of thoracic duct is also successfully reported in adult patient [7]. However, surgery for thoracic duct is successful if the effusion is chylous and lymphangiography suggests obstruction or leakage in thoracic duct [2]. In our case the pleural effusion was nonchylous and child had no ascites. Treatment with interferon-alpha or radiation therapy with a fractionated dose of 18–20 Gy has also been shown to be effective and safe [8]. Role of bisphosphonate medication for preventing osteolysis has not been investigated in children. Recently propranolol and sirolimus have been shown to improve the pleural effusion and lymphatic malformation in some cases [10, 11].

To conclude, generalized lymphatic anomaly is a rare and morbid disease in children. Presenting symptoms may be innocuous so diagnosis may be delayed. Radiological imaging therefore is very crucial for early diagnostic confirmation. Though association is rare, we suggest skeletal survey in a child with multiple subcutaneous lymphangioma. Bony lesions in GLA can be radiologically differentiated with other complex lymphatic disease of bone like GSD and KLA. Treatment is largely palliative and condition is difficult to manage in its extreme severity.
